# Clinical indications for image-guided interventional procedures in the musculoskeletal system: a Delphi-based consensus paper from the European Society of Musculoskeletal Radiology (ESSR)—part IV, hip

**DOI:** 10.1007/s00330-021-07997-5

**Published:** 2021-06-19

**Authors:** Luca Maria Sconfienza, Miraude Adriaensen, Andrea Alcala-Galiano, Georgina Allen, Maria Pilar Aparisi Gómez, Giacomo Aringhieri, Alberto Bazzocchi, Ian Beggs, Vito Chianca, Angelo Corazza, Danoob Dalili, Miriam De Dea, Jose Luis del Cura, Francesco Di Pietto, Elena Drakonaki, Fernando Facal de Castro, Dimitrios Filippiadis, Salvatore Gitto, Andrew J Grainger, Simon Greenwood, Harun Gupta, Amanda Isaac, Slavcho Ivanoski, Monica Khanna, Andrea Klauser, Ramy Mansour, Silvia Martin, Vasco Mascarenhas, Giovanni Mauri, Catherine McCarthy, David McKean, Eugene McNally, Kalliopi Melaki, Carmelo Messina, Rebeca Mirón Mombiela, Ricardo Moutinho, Marina Obradov, Cyprian Olchowy, Davide Orlandi, Raquel Prada González, Mahesh Prakash, Magdalena Posadzy, Saulius Rutkauskas, Žiga Snoj, Alberto Stefano Tagliafico, Alexander Talaska, Xavier Tomas, Violeta Vasilevska Nikodinovska, Jelena Vucetic, David Wilson, Federico Zaottini, Marcello Zappia, Domenico Albano

**Affiliations:** 1 IRCCS Istituto Ortopedico Galeazzi, Via Riccardo Galeazzi 4, 20161 Milan, Italy; 2 Dipartimento di Scienze Biomediche per la Salute, Università degli Studi di Milano, Milan, Italy; 3 Department of Medical Imaging, Zuyderland Medical Center, Sittard-Geleen, Heerlen, Brunssum, Kerkrade, the Netherlands; 4 Hospital Universitario 12 de Octubre, Madrid, Spain; 5 St Lukes Radiology Oxford Ltd, Oxford, UK; 6 University of Oxford, Oxford, UK; 7 Department of Radiology, Auckland City Hospital, Auckland, New Zealand; 8 Department of Radiology, Hospital Vithas Nueve de Octubre, Valencia, Spain; 9 Diagnostic and Interventional Radiology, Department of Translational Research and New Technologies in Medicine and Surgery, University of Pisa, Pisa, Italy; 10 Diagnostic and Interventional Radiology, IRCCS Istituto Ortopedico Rizzoli, Bologna, Italy; 11 Analytic Imaging, Edinburgh, UK; 12 Ospedale Evangelico Betania, Napoli, Italy; 13 Clinica di Radiologia EOC IIMSI, Lugano, Switzerland; 14 Epsom and St Helier University Hospitals NHS Trust, London, UK; 15 Studio MSK, Belluno, Italy; 16 Hospital Universitario Donostia, San Sebastian, Spain; 17 Dipartimento di Diagnostica per Immagini, Pineta Grande Hospital, Castel Volturno, Italy; 18 MSK Radiology Services, Heraklion, Crete Greece; 19 Hospital General Universitario de Valencia, Valencia, Spain; 20 2nd Department of Radiology, University General Hospital “ATTIKON” Medical School, National and Kapodistrian University of Athens, Haidari/Athens, Greece; 21 Cambridge University Hospitals, Cambridge, UK; 22 Mid Cheshire Hospitals NHS Foundation Trust, Cheshire, UK; 23 Leeds Teaching Hospitals, Leeds, UK; 24 School of Biomedical Engineering & Imaging Sciences, King’s College London, London, UK; 25 Guy’s and St Thomas’ Hospitals, London, UK; 26 Department of Radiology, Special Hospital for Orthopedic Surgery and Traumatology, St. Erazmo, Ohrid, North Macedonia; 27 Ss. Cyril and Methodius University of Skopje, Skopje, North Macedonia; 28 Imperial College Healthcare NHS Trust, London, UK; 29 Department of Radiology, Medical University Innsbruck, Innsbruck, Austria; 30 Oxford Musculoskeletal Radiology, Oxford University Hospitals, Oxford, UK; 31 Hospital Universitario Son Llatzer, Palma, Spain; 32 Hospital da Luz, Musculoskeletal Imaging Unit, Lisbon, Portugal; 33 AIRC, Advanced Imaging Research Consortium, Lisbon, Portugal; 34 Division of Interventional Radiology, Istituto Europeo di Oncologia IRCCS, Milano, Italy; 35 Buckinghamshire Healthcare NHS Trust, Aylesbury, UK; 36 Department of Radiology, Charité-Universitätsmedizin Berlin, Berlin, Germany; 37 Department of Radiology, Herlev and Gentofte Hospital, Herlev, Denmark; 38 Hospital de Loulé, Loulé, Portugal; 39 Sint Maartenskliniek, Department of Radiology, Nijmegen, The Netherlands; 40 Department of Oral Surgery, Wroclaw Medical University, Wroclaw, Poland; 41 Department of Radiology, Ospedale Evangelico Internazionale, Genoa, Italy; 42 Ribera Povisa Hospital, Vigo, Spain; 43 Post Graduate Institute of Medical Education & Research (PGIMER), Chandigarh, India; 44 Indywidualna Praktyka Lekarska Magdalena Posadzy, Poznan, Poland; 45 Department of Radiology, Lithuanian University of Health Sciences, Kaunas, Lithuania; 46 Institute of Radiology, University Medical Centre Ljubljana, Zaloska 7, 1000 Ljubljana, Slovenia; 47 Faculty of Medicine, University of Ljubljana, Ljubljana, Slovenia; 48 Department of Health Sciences, University of Genova, Genoa, Italy; 49 IRCCS Ospedale Policlinico San Martino, Genova, Italy; 50 Department of Radiology, AUVA Trauma Center Vienna, Vienna, Austria; 51 Radiology Dpt. MSK Unit. Hospital Clinic (CDIC), University of Barcelona (UB), Barcelona, Spain; 52 University Institute of Radiology, Clinical Center “Mother Theresa”, Skopje, Macedonia; 53 Radiology Department, Hospital ICOT Ciudad de Telde, Las Palmas, Spain; 54 Department of Medicine and Health Sciences, University of Molise, Campobasso, Italy; 55 Varelli Institute, Naples, Italy; 56 Sezione di Scienze Radiologiche, Dipartimento di Biomedicina, Neuroscienze e Diagnostica Avanzata, Università degli Studi di Palermo, Palermo, Italy; 57 Dipartimento di Oncologia e Emato-oncologia, Università degli Studi di Milano, Milano, Italy; 58 Imperial College, London, UK; 59 University of Oxford, Oxford, UK

**Keywords:** Interventional radiology, Hip, Ultrasonography, Injections, Platelet-rich plasma

## Abstract

**Objectives:**

Image-guided musculoskeletal interventional procedures around the hip are widely used in daily clinical practice. The need for clarity concerning the actual added value of imaging guidance and types of medications to be offered led the Ultrasound and the Interventional Subcommittees of the European Society of Musculoskeletal Radiology (ESSR) to promote, with the support of its Research Committee, a collaborative project to review the published literature on image-guided musculoskeletal interventional procedures in the lower limb in order to derive a list of clinical indications.

**Methods:**

In this article, we report the results of a Delphi-based consensus of 53 experts who reviewed the published literature for evidence on image-guided interventional procedures offered in the joint and soft tissues around the hip in order of their clinical indications.

**Results:**

Ten statements concerning image-guided treatment procedures around the hip have been collected by the panel of ESSR experts.

**Conclusions:**

This work highlighted that there is still low evidence in the existing literature on some of these interventional procedures. Further large prospective randomized trials are essential to better confirm the benefits and objectively clarify the role of imaging to guide musculoskeletal interventional procedures around the hip.

**Key Points:**

*• Expert consensus produced a list of 10 evidence-based statements on clinical indications of image-guided interventional procedures around the hip.*

*• The highest level of evidence was only reached for one statement.*

*• Strong consensus was obtained for all statements.*

**Supplementary Information:**

The online version contains supplementary material available at 10.1007/s00330-021-07997-5.

## Introduction

Musculoskeletal interventional procedures in the lower limb are very common. Joint, tendon, and bursal injections are routinely performed by different physicians, including radiologists, orthopedists, and physiotherapists. However, radiologists have an arrow in the quiver, namely the possibility to use imaging to guide interventional procedures [1–5]. In the hip, the most common interventional procedures focus on the treatment of joint osteoarthritis, mostly using corticosteroids and hyaluronic acid, and greater trochanteric pain syndrome (GTPS), by injecting medications or performing needling, while other bursal and tendon procedures are less frequently adopted [6–9]. Due to the anatomy of the involved structures, fluoroscopy and ultrasound are generally used to guide these procedures. Although both ultrasound and fluoroscopy may ensure higher accuracy, safety, and effectiveness of some procedures, the same concept may not be directly transferred to all types of interventions in other anatomical regions.

Furthermore, another controversial issue concerns the actual role of hyaluronic acid and regenerative medications like platelet-rich plasma (PRP) to treat musculoskeletal conditions around the hip [10, 11]. The literature is often sparse and conflicting regarding the added value of imaging guidance and type of medications to be used.

In 2019, the need of clarity on this topic led the Ultrasound and the Interventional Subcommittees of the European Society of Musculoskeletal Radiology (ESSR) to promote, with the support of its Research Committee, a collaborative project to review the published literature on image-guided musculoskeletal interventional procedures in the lower limb and to derive a list of consensus-based clinical indications, as already done for interventional procedures in the upper limb [12–14]. In this article, we report the results of a Delphi method review of evidence on published literature regarding image-guided interventional procedures around the hip listing clinical indications.

## Materials and methods

Institutional Review Board approval was not required as no patients were involved. This article was conceived as part of a collaborative project aimed to the review of image-guided musculoskeletal interventional procedures in the lower limb and to derive a list of consensus-based clinical indications. In this article, we have reported the results concerning tendon, joint, and bursal procedures around the hip. Similar to previous ESSR consensus papers [12–16], a literature-based Delphi method of review was used. This method includes a sequence of discussion rounds to assess the opinion of experts on controversial topics, drafted on the basis of the published literature, to reach a final shared agreement [17]. The AGREE II tool was employed to ensure the quality of this study [18]. Full explanation of the Delphi method, including (1) expert selection; (2) literature search, statement drafting, and level of evidence; (3) questionnaire preparation and consensus; and (4) data analysis and paper drafting are reported as supplementary material. The Oxford Centre for Evidence-based Medicine evidence levels were used [19].

## Results

1. **Ultrasound- and fluoroscopy-guided iliopsoas peri-tendinous/bursal injections with local anesthetic and corticosteroids are both safe and feasible and provide good pain relief in symptomatic patients with iliopsoas tendinopathy. This procedure may also be used to exclude the iliopsoas tendon as a cause of hip or groin pain in both arthroplasty and non-arthroplasty patients.**
  Level of evidence: 3
  Agree, *n* = 53; disagree, *n* = 0; abstain, *n* = 0. Agreement = 100%
  Iliopsoas bursa injection under ultrasound [20] and fluoroscopy guidance [21, 22] for treating iliopsoas tendinopathy or bursitis are feasible and accurate. However, there are no studies comparing the different injection techniques neither to compare the different outcome of palpation-guided and image-guided injections [23, 24]. In ultrasound-guided injections, dynamic injection with local anesthetic may be used to ensure needle placement in the bursa [25, 26]. Complications are rare, minor, and transient. Iliopsoas bursa injection may be useful to determine the origin of pain, particularly in patients with hip arthroplasty. Adler et al [25] observed that ultrasound-guided iliopsoas injections with local anesthetic and steroids after hip replacement provided relief to most patients (90%) with iliopsoas tendinosis/bursitis. Authors reported a lower success rate in non-arthroplasty patients related to the several involved conditions in hip pain, although 64% of these patients responded positively thereby confirming the diagnosis of iliopsoas tendinosis and highlighting those patients who may benefit from a surgical tendon release. Han et al showed that, in 178 patients with iliopsoas tendinopathy, ultrasound-guided iliopsoas corticosteroid and local anesthetic injection improved outcomes at 6 weeks, regardless of coexisting intra-articular hip abnormalities [26]. Patients without intra-articular hip abnormalities showed significantly greater clinical improvement than patients with intra-articular abnormalities [26]. This suggests that the presence of underlying intra-articular hip abnormalities may limit the clinical effect of iliopsoas injections in patients with iliopsoas tendinopathy, perhaps due to the pain being multifactorial. Fluoroscopy-guided iliopsoas bursa injection demonstrated a significant clinical improvement and pain reduction at 1-month post-injection in about half of the 39 patients with iliopsoas tendinopathy tested by Agten et al [21]. Sometimes more than one injection is required to relieve the symptoms [22].
2. **A single ultrasound- or fluoroscopy-guided corticosteroid and local anesthetic injection into the symphyseal cleft and/or site of abnormality detected by magnetic resonance imaging (MRI) at the rectus abdominis or adductor longus insertion may result in clinical improvement in athletes with pubalgia.**
  Level of evidence: 4
  Agree, *n* = 53; disagree, *n* = 0; abstain, *n* = 0. Agreement = 100%
  A single ultrasound-guided corticosteroid-anesthetic injection into the area of degeneration or fraying of the rectus abdominis and/or adductor longus led to clinical improvement in 12 patients with pubalgia, with all of them having returned to their preinjury activity level [27]. Fluoroscopy-guided corticosteroid-anesthetic peri-insertional injection into the symphyseal cleft and to the site of MRI-depicted abnormality resulted in clinical improvement in near 90% of 45 athletes with pubalgia, with a sustained response in 60% after 6 months [28]. An isolated superior cleft sign on MRI is more frequently associated with complete recovery. Furthermore, an initial complete response seems to be a prognostic factor, capable of predicting sustained response [28].
3. **Ultrasound-guided needling and autologous blood product injection are both safe and feasible and may improve the clinical symptoms in hamstring tendinopathy.**
  Level of evidence: 3
  Agree, *n* = 53; disagree, *n* = 0; abstain, *n* = 0. Agreement = 100%
  Percutaneous ultrasound-guided dry needling of tendons around the hip and pelvis, including hamstring tendons, may be a safe and effective treatment for tendinopathy and partial tears. In a single retrospective study [4], improvement of patient symptoms after ultrasound-guided fenestration has been described in 82% of patients with hip and pelvis tendon pathology for up to 70 days, with no complications. Several studies report the use of PRP in hamstring injuries but the limitations and variability in study design (platelet and leucocyte concentration in PRP, kit used to prepare the PRP from the autologous blood, the ideal PRP volume to administer, frequency of injection, post-injection rehabilitation care) may limit their validity. Pain and functional improvement after PRP infiltration varied from good results at 6-month follow-up [29–31] and 1-week follow-up [32] to no differences in clinical outcomes at 4- [32] and 8-week [33] follow-up. Despite limited evidence, the potential side effects of PRP are so minimal that its use for proximal hamstring pathology might be considered when other non-invasive measures are unsuccessful, but only after exhausting more established therapies.
4. **Image- and palpation-guided corticosteroid-anesthetic injections are both feasible, safe, and effective to treat greater trochanteric pain syndrome (GTPS) providing clinical improvement up to 3–6 months. Ultrasound-guided injections seem to be more effective, compared to palpation-guided injections, when performed into the greater trochanteric bursa.**
  Level of evidence: 2
  Agree, *n* = 53; disagree, *n* = 0; abstain, *n* = 0. Agreement = 100%
  Injection therapies for trochanteric bursitis with palpation guidance or image guidance are both safe and effective for relieving GTPS, resulting in a significant reduction in pain up to three months [34]. Ultrasound- and palpation-guided injections of the trochanteric bursa have also shown similar clinical results at 2-week and 6-month follow-up [35]. According to some authors, benefit in pain reduction from ultrasound-guided trochanteric bursa corticosteroid-anesthetic injection might decrease at 6 and 12 months [36, 37]. McEvoy et al [36] reported that corticosteroid injections into the greater trochanteric bursa are more effective than injections into the subgluteus medius bursa. Some authors reported that injections for gluteus medius bursitis were weakly associated with long-term pain reduction [38, 39]. One randomized double-blind placebo-controlled trial of peri-tendinous injections of glucocorticoids around the gluteal tendons showed no statistically significant outcome difference compared with normal saline injections [40]. Even though fluoroscopy can be used to successfully inject the trochanteric bursa or subgluteus medius bursa in patients with GTPS [41], in a multicenter randomized controlled trial, fluoroscopically guided injections were not associated with superior clinical outcomes at 1 month compared to palpation-guided injections alone [42].
5. **Ultrasound-guided corticosteroid injection, needling, and PRP injection for GTPS are all valuable measures to reduce pain and no clear evidence exists to define one treatment as superior to the others. PRP may have more long-lasting clinical improvement than corticosteroid injections, although high-quality evidence is still missing.**
  Level of evidence: 2
  Agree, *n* = 53; disagree, *n* = 0; abstain, *n* = 0. Agreement = 100%
  Ultrasound-guided tendon dry needling and PRP injections are valuable and relatively safe treatment strategies in patients with GTPS refractory to conservative measures. Both methods showed similar outcomes at 1 and 2 weeks and at 3 months post treatment in a single blind prospective study [43]. According to one randomized double-blind control trial [44], there is no difference in pain relief and functional improvement between ultrasound-guided PRP and corticosteroid intratendinous injection for GTPS at 2–60 days. However, patients receiving PRP achieved greater clinical improvement at 12 weeks. One prospective randomized double-blind study showed better results with corticosteroid than PRP injections in the greater trochanteric bursa [45]. Fitzpatrick et al and Begkas et al presented better and longer-lasting clinical results when treating GTPS with ultrasound-guided PRP injections compared to corticosteroid. Such results were confirmed by a systematic review [46], representing a safe and effective alternative to surgery [47]. Another prospective controlled randomized study found better and longer-lasting clinical results (at 24 weeks) in patients with GTPS treated by ultrasound-guided PRP injections compared to corticosteroid injections [48]. However, Ali et al underlined the absence of adequately powered studies providing high-quality evidence, especially when the global pathology of GTPS is considered [46]. Thus, the role of PRP in this setting still needs further investigations. Of note, based on a single prospective randomized controlled trial, physiotherapy (education and exercise) and a single ultrasound-guided injection of a corticosteroid and anesthetic for gluteal tendinopathy resulted in higher rates of global improvement and lower pain intensity than no treatment at 8 and 52 weeks [49]. Education and exercise showed better global improvement than administration of a corticosteroid and anesthetic, but with no significant difference in pain intensity, thus supporting physiotherapy as an effective management approach.
6. **Ultrasound-guided ischiogluteal bursa injections are technically feasible in cadavers but no clinical data is available.**
  Level of evidence: 4
  Agree, *n* = 52; disagree, *n* = 1; abstain, *n* = 0. Agreement = 98%
  The ischiogluteal bursa is located posterior and inferior to the ischial tuberosity and deep to the inferior portion of the gluteus maximus muscle. Ischiogluteal bursitis can present as an acute or chronic condition. The etiology includes direct trauma to the ischial tuberosity, abnormal friction between the ischial tuberosity, hamstring origin, and overlying gluteus maximus, and underlying hamstring tendinopathy [50]. Ischiogluteal bursa injection can be performed under ultrasound guidance in cadavers [51] but there are no data about indications and procedure outcome [23].
7. **Image-guided intra-articular hip injections are well-tolerated and safe procedures, which are more accurate and effective than palpation-guided injections.**
  Level of evidence: 1
  Agree, *n* = 53; disagree, *n* = 0; abstain, *n* = 0. Agreement = 100%
  Several studies reported that image-guided intra-articular hip injections improve the accuracy of intra-articular placement [52–55]. A systematic review and meta-analysis revealed that ultrasound-guided injections are significantly more accurate than palpation-guided injections (using anatomical landmarks) [56]. The accuracy of palpation-guided injections ranges from 67 to 88%, which improves to 97% when ultrasound is used. Ultrasound has also the great advantage of no ionizing radiations as compared to fluoroscopy [52, 57, 58]. Intra-articular treatment with imaging guidance is well-tolerated and safe for hip osteoarthritis patients [59]. The most common adverse effect is moderate pain during injection or lasting for a short time after injection, which usually resolves without the need for treatment [60].
8. **Positive response to image-guided diagnostic intra-articular injections with anesthetics can help confirm the intra-articular origin of hip pain.**
  Level of evidence: 3
  Agree, *n* = 53; disagree, *n* = 0; abstain, *n* = 0. Agreement = 100%
  When the pain generator site is unconfirmed, diagnostic injections may be used to diagnose a variety of underlying intra-articular hip pathologies [55, 61]. Intra-articular diagnostic injections have been reported to be a 90% reliable indicator of intra-articular abnormalities [62] and nonresponse to injection was shown to represent a strong negative predictor of surgical outcome [55]. Complete relief of hip pain following intracapsular injection of local anesthetic was reported associated with good surgical outcome following joint replacement [63, 64]. Odoom et al showed that positive response to pre-operative anesthetic injection into a hip is associated with positive prognosis after hip surgery [65]. However, for femoroacetabular impingement, injections may be more useful in non-responders [61].
9. **Image-guided corticosteroid hip injection is effective in providing short-term pain relief and can transiently improve function in patients with osteoarthritis.**
  Level of evidence: 2
  Agree, *n* = 53; disagree, *n* = 0; abstain, *n* = 0. Agreement = 100%
  Hip intra-articular corticosteroid injection may be effective in delivering short-term, but clinically significant, pain reduction in patients with hip osteoarthritis, and may also lead to transient improvement in function. The treatment effect appears to be of rapid onset with a large group of responders reported at 1-week post-injection. The magnitude of pain reduction and functional improvement decreases thereafter, although two trials reported clinically significant differences in both pain and function at 8 weeks post-injection [59, 66, 67]. Some reports show that patients with less advanced disease respond better to corticosteroids compared with patients with more advanced disease [67, 68]. A recent study on 110 patients (52 hips and 58 knees) showed that intra-articular ketorolac or triamcinolone injections provided similar improvement. Furthermore, due to its differing mechanism of action, ketorolac may not produce additional cartilage damage [69].
10. **Ultrasound-guided intra-articular hip injection of hyaluronic acid is not different for pain and function improvement from placebo, corticosteroid (at 1 and 6 months), and PRP (at 6 and 12 months) in patients with hip osteoarthritis. No different outcomes have been observed by using different hyaluronic acid formulations.**
  Level of evidence: 2
  Agree, *n* = 53; disagree, *n* = 0; abstain, *n* = 0. Agreement = 100%
  Ultrasound-guided intra-articular hip injection of hyaluronic acid does not significantly reduce pain or improve function when compared to placebo in short-term follow-up [60], with mild impact on pain and disability up to 3 months, and no difference at 6 months [70]. Brander et al [71] suggested that hyaluronic acid injection significantly improved pain scores compared to baseline at 6-months, although they did not demonstrate a superior effect over placebo. A meta-analysis found that intra-articular placebo is effective for osteoarthritis, particularly with regards to self-reported pain and functional outcome measurements, probably due to the dilution of proinflammatory cytokines by saline [72]. Regarding the different concoctions, published data showed that most hyaluronic acid formulations were not significantly different in terms of clinical and functional outcomes [73–75].

Previous studies also demonstrated the superiority of methylprednisolone over hyaluronic acid injection at 1 month (for pain and disability) and no difference at 6 months [70]. Hyaluronic acid (namely Hylan G-F 20) provided clinically meaningful improvements in pain and function, even higher than those of methylprednisolone in more advanced osteoarthritis, but with similar results in less advanced disease [68]. Nevertheless, intra-articular corticosteroid injections have been shown to provide better early outcomes, while the benefits of hyaluronic acid surpassed those of corticosteroid later in the follow-up [68]. Indeed, studies using intra-articular corticosteroids in the hip suggest a short duration of action (4–12 weeks) [67, 76, 77]. In contrast, the effects of hyaluronic acid can last up to 6 months, although recent evidence also showed no difference at this time point for hyaluronic acid and corticosteroids [60, 74, 75, 78]. A systematic review included five trials investigating the use of PRP in hip osteoarthritis, showing overall no significant differences between patients treated with PRP or hyaluronic acid alone [79].

## Discussion

Following a Delphi-based consensus, 10 statements regarding image-guided musculoskeletal interventional procedures on joints, tendons, and bursae around the hip were provided by a panel of 53 experts from the Ultrasound and Interventional Subcommittees of the ESSR. According to the results of this consensus, the evidence for procedures performed in the hip joint and trochanteric region is sparse. Specifically, statement #7 concerning the safety and higher accuracy and effectiveness of image-guided intra-articular hip injections than palpation-guided injections is the only one reaching the highest level of evidence, thus establishing the added value of imaging to guide hip injections. Interestingly, prospective randomized trials have proven the efficacy of image-guided corticosteroid hip injection to obtain short-term pain relief, with similar outcomes of ultrasound-guided intra-articular injection of hyaluronic acid with that of corticosteroid and PRP in hip osteoarthritis, allowing reaching level of evidence 2 in statements #9 and #10. Even procedures aimed to treat GTPS have reached level of evidence 2, with similar outcomes obtained using image- and palpation-guided corticosteroid-anesthetic injections, as well as with ultrasound-guided corticosteroid injection, needling, and PRP injection. On the other hand, the evidence for the remaining procedures on iliopsoas, pubic symphysis, hamstring tendons, and ischiogluteal bursa is still low. Indeed, no randomized clinical trials or well-designed prospective longitudinal trials have been published on these interventions. Notably, a strong consensus has been achieved in 100% of statements provided by the experts, with all of them having agreed on the clinical indications of these procedures, except for one disagreement concerning statement #6.

In conclusion, 10 statements concerning image-guided treatment procedures around the hip have been collected by a panel of experts from the ESSR. There remains low evidence in the existing literature on some of these interventional procedures. Further large prospective randomized trials are therefore essential to further clarify and consolidate the additional role of imaging to guiding musculoskeletal interventional procedures around the hip.

## Supplementary Information

ESM 1 (DOCX 21 kb)

## Abbreviations

ESSR: European Society of Musculoskeletal Radiology
GTPS: Greater trochanteric pain syndrome
MRI: Magnetic resonance imaging
PRP: Platelet-rich plasma

## Declarations

### Guarantor

The scientific guarantor of this publication is Luca Maria Sconfienza, MD PhD.

### Statistics and biometry

No complex statistical methods were necessary for this paper.

### Methodology

• Multicenter study
