# Supplementary material for: Clinical indications for image-guided interventional procedures in the musculoskeletal system: a Delphi-based consensus paper from the European Society of Musculoskeletal Radiology (ESSR)—part IV, hip
Source: Eur Radiol. 2021 Jun 19;32(1):551–60. doi: 10.1007/s00330-021-07997-5 (PMC8660721; doi:10.1007/s00330-021-07997-5)
Supplement: Supplementary file 1 — (DOCX 21 kb) [file 330_2021_7997_MOESM1_ESM.docx]

**Supplementary material to “Clinical indications for image guided interventional procedures in the musculoskeletal system: a Delphi-based consensus paper from the European Society of Musculoskeletal Radiology (ESSR)—part IV, hip”**

The Delphi based consensus method can be resumed as follows:

1. *Expert selection*

The panel consisted of 53 radiologists representing 16 Countries (Austria, Belgium, Denmark, Germany, Greece, India, Italy, Lithuania, New Zealand, North Macedonia, Poland, Portugal, Slovenia, Spain, The Netherlands, United Kingdom), with established experience in diagnostic and interventional musculoskeletal procedures and selected among the members of the Ultrasound and Interventional Subcommittees of the ESSR. All of them had established experience in the scientific evaluation of medical literature. This board was divided into different groups to assess specific procedures around the hip, including image-guided tendon, bursal, and joint interventions.

1. *Literature search, statement drafting, and level of evidence*

All panel members searched on the major online databases (MEDLINE, Web of Science, EMBASE, and Google), with the search terms relevant to the specific topic, including literature up to December 2020. Panel members were free to include any other papers they felt deserved to be considered, also screening all references of retrieved papers for any additional articles. On the basis of the retrieved evidence, all groups listed the statements in draft form for their assigned topic and levels of evidence were provided to each statement according to the criteria of the Oxford Center of Evidence-Based Medicine in 2011 [1]. To this purpose, we used the section “Does this intervention help? (Treatment Benefits)”: systematic review of randomized trials or n-of-1 trials are classified as level 1, randomized trial or observational study with dramatic effect are classified as level 2, non-randomized controlled cohort/follow-up study are classified as level 3, case-series, case-control studies, or historically controlled studies are classified as level 4, and mechanism-based reasoning is classified as level 5.

1. *Questionnaire preparation and consensus process*

The coordinator revised the drafted statements and sent an online questionnaire (Google Forms, Google LLC) with an access link that was disseminated via email to the entire board of experts. In this form, each member was asked to agree, disagree, or abstain with the drafted statements. Also, they could provide their comments on each statement, with all members having been blinded to the comments provided by others. All answers and comments were automatically collected in an electronic spreadsheet (Microsoft Excel, Microsoft). After the first round of review, the coordinator evaluated all answers and comments and edited the drafted statements accordingly. Then, a second round with the same modalities was done. After a second round, persisting conflicts were solved or confirmed via targeted emails.

1. *Data analysis and paper drafting*

At the end of the Delphi process, the consensus opinion was obtained for all statements. The consensus was considered as strong if more than 95% experts agreed, while it was considered as broad when more than 80% but less than 95% agreed on the statement [2]. Last, the article was drafted according to the results of the Delphi-based consensus and shared via email among all panel members for final approval.

**References to supplementary material**

1. Oxford Center of Evidence Based Medicine. OCEBM Levels of Evidence 2016 v. 2.1. 2016 <https://www.cebm.net/wp-content/uploads/2014/06/CEBM-Levels-of-Evidence-2.1.pdf> Accessed on 3 July 2019
2. Săftoiu A, Gilja OH, Sidhu PS, et al (2019) The EFSUMB Guidelines and Recommendations for the Clinical Practice of Elastography in Non-Hepatic Applications: Update 2018. Ultraschall Med. doi: 10.1055/a-0838-993
